# Clinical and cost-effectiveness of guided internet-based interventions in the indicated prevention of depression in green professions (PROD-A): study protocol of a 36-month follow-up pragmatic randomized controlled trial

**DOI:** 10.1186/s12888-019-2244-y

**Published:** 2019-09-09

**Authors:** Lina Braun, Ingrid Titzler, David Daniel Ebert, Claudia Buntrock, Yannik Terhorst, Johanna Freund, Janika Thielecke, Harald Baumeister

**Affiliations:** 1 Department of Clinical Psychology and Psychotherapy, Institute of Psychology and Education, University of Ulm, Ulm, Germany; 2 Department of Clinical Psychology and Psychotherapy, Friedrich-Alexander-University of Erlangen-Nuremberg, Erlangen, Germany; 3 GET.ON Institute, Hamburg, Germany; 4 Department of Clinical, Neuro- & Developmental Psychology, VU University Amsterdam, Amsterdam, Netherlands

**Keywords:** Prevention, Depression, Internet- and mobile-based interventions, Green professions, RCT

## Abstract

**Background:**

People in green professions are exposed to a variety of risk factors, which could possibly enhance the development of depression. Amongst possible prevention approaches, internet- and mobile-based interventions (IMIs) have been shown to be effective and scalable. However, little is known about the effectiveness in green professions. The aim of the present study is to examine the (cost-)effectiveness of a tailored IMI program for reducing depressive symptoms and preventing the onset of clinical depression compared to enhanced treatment as usual (TAU+).

**Methods:**

A pragmatic randomized controlled trial (RCT) will be conducted to evaluate a tailored and therapeutically guided preventive IMI program in comparison to TAU+ with follow-ups at post-treatment (9 weeks), 6-, 12-, 24-, and 36-months. Entrepreneurs in green professions, collaborating spouses, family members and pensioners (*N* = 360) with sufficient insurance status and at least subthreshold depression (PHQ-9 ≥ 5) are eligible for inclusion. Primary outcome is depressive symptom severity (QIDS-SR16). Secondary outcomes include incidence of depression (QIDS-SR16), quality of life (AQoL-8D) and negative treatment effects (INEP). A health-economic evaluation will be conducted from a societal perspective. The IMI program is provided by psychologists of an external service company and consists of six guided IMIs (6–8 modules, duration: 6–8 weeks) targeting different symptoms (depressive mood, depressive mood with comorbid diabetes, perceived stress, insomnia, panic and agoraphobic symptoms or harmful alcohol use). Intervention choice depends on a screening of participants’ symptoms and individual preferences. The intervention phase is followed by a 12-months consolidating phase with monthly contact to the e-coach.

**Discussion:**

This is the first pragmatic RCT investigating long-term effectiveness of a tailored guided IMI program for depression prevention in green professions. The present trial builds on a large-scale strategy for depression prevention in green professions. The intended implementation of the IMI program with a nationwide rollout has the potential to reduce overall depression burden and associated health care costs in case of given effectiveness.

**Trial registration:**

German Clinical Trial Registration: DRKS00014000. Registered on 09 April 2018.

## Background

Major depressive disorder (MDD) is a highly prevalent disorder in the general population: a 12-month prevalence of MDD amounts to 6.7% [1], while lifetime prevalence is estimated at 10.6 to 19.8% [2]. MDD is associated with substantial functional impairment [3, 4] and has been identified as a leading cause of global disease burden [5]. In addition to the high individual burden, the socioeconomic costs associated with MDD and its comorbid physical and psychiatric disorders due to medical use, absenteeism, loss of productivity at work and suicide, are enormous [6].

An estimation of prevalence rates in the specific target group of green professions in Germany has yet to be conducted. However, people in green professions are exposed to a variety of risk factors, which might increase vulnerability for developing MDD. Risk factors include financial pressure, high administrative workload, long working hours, high stress levels, part-time jobs off the farm, health problems and preceding work accidents [7, 8], as well as continuous exposure to pesticides [8–10]. In addition, an increased risk of suicide in the agricultural sector was reported across different countries e.g. in England and Wales [11, 12], the USA [13–15], Australia [16, 17], and Brazil [9]. Based on given risk factors and risk of suicide, preventive measures are highly indicated.

Psychological interventions for subthreshold depression as indicated prevention have been shown to significantly reduce depressive symptom severity [18]. Additionally, such interventions might even prevent the onset of major depression [18, 19]. Thus, preventive interventions can have a substantial role in reducing overall depression burden in green professions.

Although face-to-face psychotherapy represents an effective treatment option, impact of such on-site offers is restricted. MDD is still an underdiagnosed and undertreated condition with only approximately 50% of persons with 12-month MDD receiving some kind of treatment, and only approximately 20% of affected persons receiving an appropriate treatment concordant with treatment guidelines [4]. In Germany, availability of on-site psychotherapy is restricted by long waiting times and is furthermore lower in rural areas than in urban areas [20].

Internet- and mobile-based interventions (IMIs) have already shown to be effective in reducing subthreshold depression [21–25] and thus, pose a suitable alternative treatment option to on-site psychotherapy. While short-term effectiveness of IMIs regarding depression severity at post-treatment is well established, studies on long-term effectiveness are scarce and yield inconsistent evidence [26].

IMIs constitute a promising approach for providing an effective and easily accessible prevention offer in routine health care [27, 28]. As IMIs can be used independently of time and place, they are easily accessible even in rural areas and have the potential to increase acceptance and utilization of psychological interventions [29]. Currently, little is known about acceptance, effectiveness or utilization of IMIs in the specific target group of green professions.

To improve the delivery situation in rural areas, and specifically for the target group of green professions, this study will be conducted in the framework of a national depression prevention program for people in green professions, implemented by the Social Insurance for Agriculture, Forestry and Horticulture (SVLFG, http://www.svlfg.de/). The SVLFG currently establishes a comprehensive prevention model project (“With us in balance”) for their insurees with the offered services of guided IMIs, personalized tele-based coaching as well as on-site prevention group workshops, provided by different service providers in the health care sector. Parallel to a nationwide rollout, the single internet- or telephone-based prevention offers will be evaluated in randomized controlled clinical trials (RCTs) in order to establish evidence on the clinical and cost-effectiveness. Eligible for this trial are policyholders of the SVLFG who are entrepreneurs, family members, spouses or have retired in one of the three sectors of agriculture, forestry or horticulture (referred to as “green professions”).

In the present study, IMIs for the prevention of depression provided by the health care company GET.ON will be evaluated regarding effectiveness and cost-effectiveness. GET.ON institute offers scientifically evaluated IMIs for different mental health problems (depressed mood in general or in the context of diabetes mellitus, insomnia, stress, panic and agoraphobic symptoms and harmful alcohol use). Even though these IMIs differ regarding their focus on mental health problems, they will be evaluated as part of a depression prevention program. The underlying idea follows the rational of a transdiagnostic approach: Transdiagnostic research has already yielded promising evidence that treatment protocols based on transdiagnostic maintaining factors and treatment concepts can lead to symptom reduction comparable to disorder-specific treatments [30, 31]. This already indicates that psychological interventions do not necessarily need to be disorder-specific and labeled accordingly to achieve symptom alleviation.

Especially in prevention context, it is desirable to reach and provide preventive interventions for a large population sample. Low adherence and high dropout rates [32, 33] restrict impact of IMIs. Treatment credibility and treatment motivation have been shown to predict treatment adherence [34, 35]. Thus, measures to increase motivation or treatment credibility are highly desirable, as they improve participation in preventive interventions and reduce dropout rates of participants [36–39]. The (cost)effectiveness of this tailored and guided prevention approach is especially interesting, as it leaves room for greater emphasis on personal interest and treatment motivation in the selection procedure of an appropriate intervention. Further, stigma against depression can still be a common issue in rural areas [40, 41]. This patient-centered and complaint-based approach also has the potential to improve the uptake of IMIs especially for depression prevention, by avoiding the exclusive label of “depression” and potentially associated stigma.

### Objective and research question

The aim of the present study is to investigate whether a tailored IMI program targeting a variety of specific complaints associated with clinical depression is useful in reducing depressive symptoms and preventing onset of clinical depression as compared to enhanced treatment as usual (TAU+) within a 36-month follow-up period. We propose, that depressive symptomology will be reduced to a greater extent in the intervention group (e.g. IMI program) than in the control condition (e.g. TAU+) post-treatment (T1). The following research questions will be investigated in this pragmatic randomized controlled trial over a follow-up period of 36-months:

- Is the IMI program effective in reducing depressive symptom severity compared to TAU+?
- Is the IMI program effective in preventing the onset of clinical depression as compared to TAU+?
- Is the IMI program effective in reducing various mental health outcomes (e.g. stress, anxiety, insomnia) compared to TAU+?
- Is there a differential or rather an undifferentiated, general effect of specific IMIs on different mental health outcomes?
- Is the IMI program superior in terms of cost-effectiveness and QALY health gains compared to TAU+?
- Which variables moderate and mediate the effects of the IMI program?
- What is the level of intervention satisfaction, adherence and acceptance to the IMI program?
- Are there negative effects of the IMI program?

## Methods

### Study design

A two-armed pragmatic randomized controlled trial (RCT) will be conducted comparing the clinical and cost-effectiveness of a tailored, guided IMI program to a control group (CG) receiving enhanced treatment-as-usual (TAU+). Primary and secondary outcomes will be assessed over a period of 36 months. Assessments will take place at baseline (T0), post-treatment (nine weeks, T1), as well as at 6-month (T2), 12-month (T3), 24-month (T4) and 36-month (T5) follow-up.

This clinical trial has been approved by the ethics committee of University of Ulm (No. 454/17) and will be reported in accordance with the Consolidated Standards of Reporting Trials (CONSORT) Statement 2010 and the extension for reporting pragmatic trials [42–44] as well as the guidelines for executing and reporting internet intervention research [45]. This trial protocol was created according to SPIRIT guidelines [46, 47]. This study is registered in the German clinical trial register under DRKS00014000.

### Participants & procedure

#### Inclusion and exclusion criteria

We will include a) agriculturists, foresters and horticulturists with sufficient insurance status of a social insurance company (SVLFG) in Germany. The study is accessible for entrepreneurs, collaborating spouses and family members as well as pensioners who do no longer contribute to the production process with sufficient insurance status. Participants are required to b) be age 18 or above, c) show an indication for at least subthreshold depression (PHQ-9 ≥ 5), d) have internet access, and e) be willing to give informed consent. Participants will be excluded a) if they are currently receiving psychotherapy (self-report), b) if they are not able to distance themselves from suicidal ideation (not able to sign a non-suicide contract), and c) if they have comorbid chronic pain symptomology (Chronic Pain Grade Questionnaire (CPG) ≥ Grade II + chronic pain longer than six months) and prefer to participate in the parallel clinical trial PACT-A on improving pain related distress in people with chronic pain (Terhorst Y, Braun L, Titzler I, Buntrock C, Freund J, Thielecke J, et al.: Effectiveness and cost-effectiveness of an acceptance and commitment therapy to improve chronic pain-related disability in green professions (PACT-A): study protocol of a 36-month follow up pragmatic randomized controlled trial, in preparation).

#### Recruitment

**Fig. 1:**
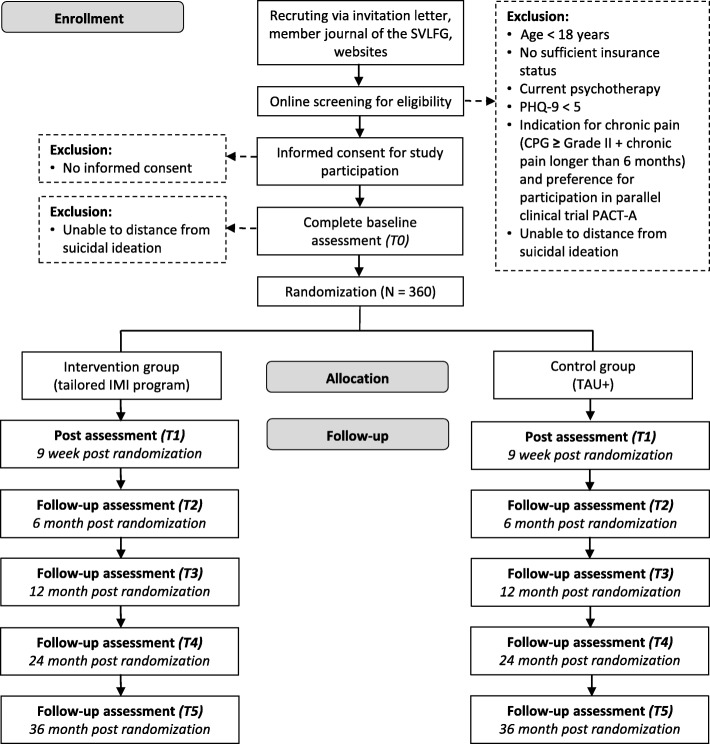
Overview of study procedure

This clinical trial is embedded in a large-scale mental health initiative of the SVLFG with the aim to improve the prevention of mental health problems in agriculturists, foresters and horticulturists. Participants are recruited via several articles in the members-journal (quarterly circulation 1.3 million), postal mailings as well as information on associated websites. Since interested persons are provided with various ways of contact (via postal return element, fax, e-mail, telephone) and an alternatively direct access to the screening assessment (via QR-Code and URL), a low-threshold access into the study is provided. Recruitment started in January 2018 and was concluded in April 2019 (Fig. 1).

#### Assessment of eligibility and randomization

People are screened for eligibility via an online screening questionnaire. Screening includes online questionnaires to assess the presence and the severity of depressive (PHQ-9 ≥ 5) and chronic pain symptomology (CPG ≥ Grade II + chronic pain longer than six months), as well as possible suicidal ideation. Online questionnaires include self-report of age, insurance status, and current psychotherapy. If the eligibility criteria are fulfilled, applicants will receive an online information letter including detailed information about study procedure and informed consent. They will be informed that they can withdraw from the intervention and/or study at any time without any negative consequences. After giving informed consent, participants will enter the study and complete baseline assessment (T0).

Subsequently, participants will be randomly allocated to intervention or control condition and will be informed about group membership via e-mail. Randomization will take place at an individual level. Randomization will be conducted by a person who is blind regarding all processes within the intervention. Whereas blinding of participants is not possible, data collectors are blinded regarding group membership. Group membership is only known by the persons administering allocated treatments to participants. The automated web-based programme sealed envelope (www.sealedenvelope.com) is used to perform permuted block randomization with randomly arranged block sizes (8, 10, 12) and an allocation ratio of 1:1.

Written informed consent for participation in online-assessments will also be obtained from e-coaches. No eligibility criteria are applied by the study team for selection and assignment of e-coaches. E-coaches are employed, assigned, guided and supervised by the GET.ON institute regarding their task of counselling the study participants.

### Intervention

#### Intervention condition

Participants in the intervention group (IG) will be transferred to the service provider GET.ON to get access to a tailored and guided IMI program with the option of conducting one of six available IMIs. The IMI program consists of a 3-step intervention entailing (1) *psycho-diagnostic assessment*, (2) *participatory selection of a suitable (guided) IMI* and (3) *access to IMI*. The IMI program will be carried out by the external service company GET.ON without involvement from the study team. Thus, the GET.ON institute is responsible for providing an adequate care of the participants against the background of the psycho-diagnostic assessment and the information they receive in the course of patient contact. This includes for example the handling of adverse events in the course of e-coaching. The study team evaluates in this setting the standard procedure of the GET.ON institute for the allocation of the different trainings and the treatment of participants in the course of the IMI program.

1. The *psycho-diagnostic assessment* is based on a selection of self-report questionnaires measured as part of baseline assessment (T0). Relevant baseline assessment values are transmitted electronically to GET.ON for each participant to allow for psycho-diagnostic assessment based on allocation rules applied in supply routine of GET.ON. Each participant is assigned to a personal e-coach after signing in to the intervention platform. E-coaches are trained psychologists, who are in training for a further qualification in psychotherapy and supervised by an experienced psychological therapist.
2. Participants are contacted via telephone or private message function on the intervention platform by their personal e-coach for *participatory selection of a suitable (guided) IMI.*

- The IMI program entails six different IMIs with the aim to reduce subthreshold depression by focusing on depressed mood in general (*GET.ON Mood Enhancer*) or in the context of a comorbid diabetes mellitus (*GET.ON Mood Enhancer Diabetes*), by improving sleep quality (*GET.ON Recovery*), or by reducing subjective stress load (*GET.ON Stress*), panic symptoms (*GET.ON Panic*), or harmful alcohol use (*GET.ON Be smart - Drink less*). These interventions have already been shown to effectively reduce depressive symptom severity as primary [23, 25, 48, 49] or secondary outcome [24, 50–55], (Ebenfeld L, Lehr D, Ebert D, Kleine Stegemann S, Funk B, Riper H, et al.: Treating panic on the go: results of a randomized controlled trial evaluating a hybrid online-training for panic and agoraphobia symptoms, in preparation) to prevent the incidence of MDD [24] in other target groups. Moreover, health economic outcome evaluations alongside randomized trials indicate a promising cost benefit [56–59].
  The type of the IMI is chosen depending on psycho-diagnostic assessment as well as individual participant needs, personal preference and problem areas, which are reported in the course of the conversation.

(3) Directly afterwards *the participants will receive access to the selected IMI*. The IMIs each consist of 6–8 lessons with a duration of 30–60 min. Participants are advised to do one lesson per week. Thus, completion of the intervention is expected to take 6–8 weeks. Depending on the individual pace of the participant, the completion of the intervention can differ. After each lesson, participants get an individualized feedback by their personal e-coach. Guidance is employed to improve adherence of participants and decrease dropout [60]. Depending on the participant’s preference, feedback is given either by telephone or through the internal messaging function of the system in which the online-training is implemented. Afterwards the next lesson is unlocked for the use. In exceptional cases, it is possible to switch the training directly if the assigned tailored intervention is perceived to be unsuitable by the participant.

The IMIs are based on principles of cognitive behavioural therapy (*GET.ON Mood Enhancer*, *GET.ON Mood Enhancer Diabetes, GET.ON Recovery, GET.ON Panic, GET.ON Be smart - drink less)* and on the transactional model of stress and coping [61] (*GET.ON Stress).*

- *GET.ON Mood Enhancer* aims to reduce depressed mood by promoting the uptake of positive activities and the learning of problem-solving strategies [62].
- *GET.ON Mood Enhancer Diabetes* is an adaptation of *GET.ON Mood Enhancer* and aims to reduce depressed mood in the context of diabetes mellitus [63].
- *GET.ON Recovery* is directed towards persons with clinical insomnia complaints and aims to prevent depression by improving participants’ sleep quality, especially through modifying coping behaviour regarding rumination and worrying. This is achieved by assignments such as keeping a diary about ruminating thoughts [64].
- *GET.ON Stress* aims to reduce depressive symptomology by addressing chronic stress and reducing stress-related complaints. The training instructs to apply problem- and emotion-oriented stress management strategies based on the distinction between solvable and unsolvable problems [65].
- *GET.ON Panic* aims to reduce depressive symptoms by addressing panic symptoms and agoraphobia. Participants are instructed to perform in-vitro and in-vivo exposition. Further, the training comprises information and exercises for coping with negative automatic thoughts and for establishing positive thought processes [66]. If this training is assigned, participants are requested by their consecutive e-coach to clarify their physical symptoms with their GP in advance to rule out the presence of physical illnesses (e.g. cardiovascular diseases). The consultation of the GP is the responsibility of the participant and will not be controlled.
- *GET.ON Be smart - drink less* was developed to prevent depression by reducing harmful alcohol use in participants, who do not yet meet clinical criteria for alcohol dependence. This training aims to identify emotions and situations associated with alcohol consumption by keeping an alcohol-diary. Further, alternative exercises for actions for coping with negative emotions and problems are included [67]. This training will not be assigned to participants with a hazardous drinking level score of > 20 on AUDIT. In case of AUDIT > 20, participants will receive access to a different training of the portfolio which is most fitting to the complaints and be made aware of their hazardous drinking level by their consecutive e-coach. If interested, the e-coach will provide them with a list of addresses, which they can turn to.

The IMIs are accessible on an online-based intervention platform via either computer (e.g. desktop PC, laptop) or mobile device (e.g. tablet, smartphone). The IMIs contain psycho-educative text material, exercises, and testimonials of exemplary personas, who accompany the participants throughout the intervention. Additionally, interactive elements in form of auditory material and videos clips are included. Audio clips for example are used to convey relaxation techniques, while video clips often serve to give a comprehensible overview about theoretical concepts by experts. Since a focal point is the transfer of content and strategies learned into every-day life, many application examples, instructions and exercises, as well as homework assignments for practising between lessons are included. The content of the online-trainings has been specifically adapted to the target population by inclusion of fitting picture and video material, as well as exemplary testimonials and fictional personas living and working in the agricultural setting. These personas describe the struggle with everyday problems and challenges in green professions, e.g. generational conflicts or handing over of the company to the younger generation.

Subsequent to the completion of the IMI, participants are followed up for 12 months by their individual e-coach to consolidate the treatment effect. During this phase, participants are contacted once a month through telephone or the internal messaging system by their personal e-coach to review their current state, report problems, and give account regarding the transfer of strategies learned into the daily routine.

#### Control condition

Both participants of the IG and the CG will have unrestricted access to treatment-as-usual (TAU). Participants of the CG will receive an online information letter with psycho-educative information about stress, depression and chronic pain. Additionally, a link to an online freely available audio CD with information about stress reduction is provided, as well as a link to a database with different health services and information about further treatment options in standard care (enhanced treatment as usual (TAU+) for the CG).

TAU will not follow a standardized protocol, however, health care consumption will be measured with the TiC-P [68] (see measures below). Hence, an accurate description of TAU can be provided.

### Sample size/power calculation

In this trial, the effectiveness of the IMI program in reducing depression severity is compared to TAU+. The estimation of sample size was based on a meta-analysis by Zhou and colleagues (2016) [26], who reported an effect size of *SMD* = 0.46 [95%-CI: 0.22–0.70] for IMIs in the reduction of subclinical depressive symptoms. In this meta-analysis mostly waitlist control groups where included. Since comparison of IG with TAU+ presumably yields a smaller effect size than comparison with waitlist control group [69], we assumed a conservative effect size of *d* = 0.35 for the present trial. An effect size of *g* = 0.35 [95%-CI: 0.23–0.47] was also reported in a meta-analysis as the effect of psychotherapy on reduction of depressive symptom severity in subthreshold depression with more than a half of the included studies applying care as usual in the CG [18]. Based on prior findings [23–25, 48–55], (Ebenfeld L, Lehr D, Ebert D, Kleine Stegemann S, Funk B, Riper H, et al.: Treating panic on the go: results of a randomized controlled trial evaluating a hybrid online-training for panic and agoraphobia symptoms, in preparation) and the above stated hypothesis, a one-sided t-test (α = 0.05; 1-β = 0.90) with a ratio of 1:1 (IG:CG) is used for power calculation. Power calculation was conducted with G*Power (Version 3.1.9.2) and resulted in an overall sample size of 282 participants. According to a meta-analysis approximately 28% percent of dropout occurred across a sample of studies evaluating efficacy and effectiveness of online-based interventions for depression with therapeutic support [69]. To compensate for an expected dropout of 28%, the present study aims at an overall sample size of 360 participants.

### Assessments

All assessments will be conducted online. For an overview of instruments at screening, baseline (T0), post-treatment (T1), and follow-up assessments (T2-T5), see Table 1. Participants will receive 10€ for completion of each follow-up assessment, starting with T1.

**Table 1:** Overview of the assessments

Instruments	Aim	Time of measurement						
		Screening	T0	T1	T2	T3	T4	T5
Screening Instruments								
PHQ-9	Patient Health Questionnaire	✔						
CPG	Chronic Pain Grade Questionnaire	✔						
Chronicity of Pain	Pain longer for 6 months (yes/no)	✔						
Primary Outcome								
QIDS-SR16	Quick Inventory for Depressive Symptomatology		✔	✔^a^	✔	✔	✔	✔
Secondary Outcomes								
Adapted items from CIDI 3.0, CIDI-SC, and Epi-Q Screening Survey	Prevalence of major depression and bipolar disorder		✔	✔	✔	✔	✔	✔
PSS-10	Perceived Stress Scale		✔	✔	✔	✔	✔	✔
ISI	Insomnia Severity Index		✔	✔	✔	✔	✔	✔
GAD-7	Generalized Anxiety Disorder		✔	✔	✔	✔	✔	✔
PAS	Panic and Agoraphobia Scale		✔	✔	✔	✔	✔	✔
MPI	Multidimensional Pain Inventory		✔	✔	✔	✔	✔	✔
NRS	Pain Intensity (0–10)		✔	✔	✔	✔	✔	✔
AUDIT-10	Alcohol use disorder identification test		✔	✔	✔	✔	✔	✔
MBI-GS	Maslach-Burnout-Inventory (subscale emotional exhaustion)		✔	✔		✔		
AQoL-8D	Quality of life		✔	✔	✔	✔	✔	✔
SPE	Subjective prognosis of employment		✔	✔	✔	✔	✔	✔
Cost measurement								
TiC-P	Utilisation of health services, work-related productivity		✔	✔	✔	✔	✔	✔
Intervention-related Outcomes								
WAI-SR^b^, WAI-SRT^c^	Therapeutic relationship			✔		✔		
TAI-OT^b^	Technological alliance			✔	✔			
CSQ-I	Patient satisfaction			✔				
INEP^b^	Inventory of negative effects in psychotherapy			✔	✔	✔		
Negative effects^b^	Negative effects in psychotherapy			✔	✔	✔		
Other assessments								
Other questions	Socio-demographics		✔					
	Predictors for development of major depression		✔					
Diabetes	Diabetes diagnosis (yes/no)		✔					
BDI-II	Suicidality-Item	✔	✔	✔	✔	✔	✔	✔

^a^Primary outcome is the standardized mean difference between intervention and control group at T1. QIDS-SR16 will also be assessed at T0 and T2-T5

^b^Recorded in intervention group only

^c^Recorded in e-coaches only

*T0* Baseline, *T1* 9 weeks, *T2* 6 months, *T3* 12 months, *T4* 24 months, *T5* 36 months

#### Screening

The preliminary screening assesses sociodemographic variables (age, gender, employment relationship, insurance status) and information about various recruitment channels.

The German Version of the Patient Health Questionnaire (PHQ-9) [70] is administered as a depression screening inventory to detect subthreshold depression (PHQ-9 ≥ 5). The PHQ-9 consists of 9 items on a 4-point-scale with a rating scale ranging from 0 to 3 (0 = “not at all”, 1 = “several days”, 2 = “more than half the days”, 3 = “nearly every day”). Each item covers one symptom criterion domain of MDD. Additionally, an item is included to register severity of daily life limitations associated with depressive symptoms. The computerized version of the PHQ-9 (α = 0.88) shows an equally high internal consistency as the paper-pencil version (α = 0.89) [71]. Item 9 of BDI-II [72] will be applied to screen for suicidal ideation.

The Chronic Pain Grade Scale (CPG) [73] is applied to screen for chronic pain syndrome in combination with an additional item to measure chronicity of pain. The CPG grades pain severity by using the two dimensions “pain intensity” and “disability”. Severity of pain is categorized into the four hierarchical classes “Grade I” to “Grade IV”. The German Version of the CPG [74] showed overall a good internal consistency (α = .82). The German Version was adapted for this study to evaluate overall pain over the last 6 months.

## Outcome measurements

### Primary outcome

#### Depressive symptom severity at post-treatment (T1)

The primary outcome is depressive symptom severity at post-treatment (T1), assessed with the German Version of the Quick Inventory Depressive Symptomology (QIDS-SR16) [75]. The 16-item self-report inventory covers all nine DSM-5 symptom criterion domains of MDD. The QIDS-SR16 is characterized by high internal consistency of α = .86 [75]. The items are rated on a 4-point-scale ranging between 0 and 3. The total score ranges between 0 and 27, with a higher score indicating higher depressive symptom severity. Whereas a score between 0 and 5 indicates normal health status, scores between 6 and 10 indicate mild depressive symptomology, between 11 and 15 moderate, between 16 and 20 severe and scores greater than 20 indicate very severe depressive symptomology. Since the QIDS-SR16 is sensitive to symptom change and provides very similar results compared to longer clinical ratings like HAM-D17, HAM-D21, and HAM-D24 [75], this inventory will be administered to monitor changes in depressive symptom severity.

### Secondary outcomes

#### Depressive symptom severity at follow-up (T2 – T5)

Depressive symptom severity will be measured over a period of 36 months at 6-month (T2), 12-month (T3), 24-month (T4) and 36-month (T5) follow-up with QIDS-SR16.

#### Depression response

Clinical significance of depression response will be determined with QIDS-SR16 scores according to Jacobson and Truax [76].

#### Onset of clinical depression and bipolar disorder

Additionally, QIDS-SR16 will be used to classify participants regarding presence of clinical depression at all measurement points (T0-T5). A comparison of QIDS-SR16 scores to current and lifetime diagnosis based on Structured Clinical Interview for DSM-IV-TR Axis I Disorders (SCID) as measure criterion showed that QIDS-SR16 is a reliable screening instrument for diagnosis of clinical depression [77]. Cut-off scores of 13 and 14 yielded best results for sensitivity (76.5%) and specificity (81.8%) leading to correct classification of over 80% of participants [77]. For this study, a score ≥ 13 is defined as cut-off to identify possible cases of clinical depression for all measurement points within the 36-month follow-up period.

Further, Items adapted from the Composite International Diagnosis Interview version 3.0 (CIDI 3.0) and Screening Scales (CIDI-SC) [78], and the Epi-Q Screening Survey [79] as applied in the WHO World Mental Health Surveys International College Student Project [80] are used to assess presence of major depressive episode (MDE) and bipolar disorder (BPD) at all measurement points. Items recording onset and frequency of MDE and BPD episodes are included.

#### Perceived Stress

The 10-item version of the perceived stress scale (PSS-10) [81, 82] will be used to measure the perception of stress in participants. The scale particularly assesses how “unpredictable, uncontrollable, and overloading respondents find their lives” [83]. For the PSS-10 satisfactory internal consistencies from α = .78 to α = .91 were reported [83]. In a representative German community sample, a Cronbach’s alpha of .84 was reported for PSS-10 [84]. The PSS-10 was modified to address perception of stress load in the last week instead of the last month, because post-assessment (T1) is 9 weeks after randomization and duration of online-training lies between 6 to 8 weeks.

#### Insomnia Severity

Insomnia Severity will be measured using the Insomnia Severity Index (ISI) [85]. The ISI is a brief self-report scale composed of seven items to identify clinical insomnia. The questionnaire has been validated in clinical and community samples and is characterized by high internal consistency (α = 0.90 to 0.92) and adequate discriminative validity of the individual items [86, 87]. The German version of the ISI was examined in three cross-sectional studies in different target groups [88]. Cronbach’s Alpha was shown to be satisfactory in all three samples (α = .76 in adolescents, α = .77 in young adults, α = .81 in adult workers) [88].

#### Generalized Anxiety Disorder

The GAD-7 [89] will be used as a short self-report measure to assess likelihood of Generalized Anxiety Disorder. In the United States a high internal consistency of α = .92 was reported for the seven items of GAD-7. In a German sample, an internal consistency of α = .89 was found [90]. Overall, the GAD-7 is a valid and reliable instrument to screen for Generalized Anxiety Disorder [89, 90].

#### Panic and Agoraphobia

The severity of panic and agoraphobic symptoms will be assessed with the self-report version of the panic and agoraphobia scale (PAS) [91, 92]. The 13-item scale comprises five subscales, assessing panic attacks, agoraphobic avoidance, anticipatory anxiety, daily life limitations and health concerns. Additionally, the PAS contains an extra item for assessing unexpectedness versus expectedness of panic attacks, which is not included in the calculation of the total score [91]. The PAS was found to have a high internal consistency of α = .88 [91].

#### Pain intensity and pain associated disability

Assessment instruments for measurement of pain intensity and pain associated disability were selected under consideration of the recommendations of the Initiative on Methods, Measurement and Pain Assessment in Clinical Trials (IMMPACT) [93, 94].

The Multidimensional Pain Interference Scale (MPI) [95, 96] is administered to measure the degree of pain-associated disability regarding all-day activities. The German version of the MPI consists of 10 items rated on a 7-point scale. The MPI is characterized by excellent internal consistency (Cronbach’s alpha = .94) and good retest reliability (r = .78). Additionally, pain intensity will be recorded using an 11-point numerical rating scale from 0 (“no pain”) to 10 (“pain as bad as you can imagine”). This scale will be applied to rate the worst, least, average and current pain participants experienced during the last seven days.

#### Alcohol consumption

The Alcohol Use Disorder Identification Test (AUDIT) [97] will be applied to screen for harmful alcohol use. The AUDIT has been validated in six different countries and therefore is cross-nationally applicable [98, 99]. The 10-item self-report questionnaire measures a unidimensional construct with adequate internal consistency ranging between α = .8 and α = .83 [100, 101]. Validation of the AUDIT in a German general practice sample showed high retest reliability (ICC = .95) and adequate validity [102]. In the present study, the German Münster Version of the AUDIT following S3 guideline was applied (http://auditscreen.org/cmsb/uploads/audit-german-m-nster.pdf).

#### Emotional Exhaustion

Emotional exhaustion as the basic dimension of the burnout construct will be measured with a subscale of the Maslach Burnout Inventory (MBI-GS) [103]. In the present study, a German translation by Cillien and colleagues (2006) [104] was used. The 5-item subscale was applied to assess emotional exhaustion.

#### Quality of Life

Health-related quality of life will be assessed with the self-report questionnaireassessment of Quality of Life (AQoL-8D). This questionnaire consists of 35 items, covering the three physical dimensions “independent living”, “pain”, and “senses”, as well as the five psycho-social dimensions “mental health”, “happiness”, “coping”, “relationships”, and “self-worth” [105]. The AQoL-8D is characterised by a high Cronbach’s Alpha of 0.96 and good psychometric properties [105] and will be applied for cost-utility analyses.

#### Work capacity

Work capacity will be measured with the German version of the Subjective Prognostic Employment Scale (SPE) [106]. The SPE is a validated short self-report scale composed of 3 items with high internal consistency (Guttman scaling: rep = .99) for assessment of subjective endangerment and prognosis of work capacity [106].

### Cost measures

The provider (GET.ON institute) will provide information about the intervention costs, including costs pertaining initial phone calls by psychologists, documentation and coaching (e.g. individual written feedback) provided by qualified psychologists, technical support, server for hosting the interventions and overhead. Cost evaluation will be based on the German version of the Dutch cost questionnaire “Trimbos Institute and Institute of Medical Technology Questionnaire for Costs Associated with Psychiatric Illness” (TiC-P) [68]. In this self-report questionnaire, the usage of health care services (e.g. general practice services, intake of medications, sessions with psychotherapists or psychiatrists) and productivity loss (e.g. hospital days, absenteeism, presentism) is assessed. We will follow the human capital approach to value productivity losses [107]. The questionnaire was specifically adapted to the population of agriculturists, forester and horticulturists. A list of unit cost prices will be used to compute the total health care costs on a per-participant basis [108].

### Intervention-related outcomes

#### Experience of Working and Technological Alliances

The short version of the Working Alliance Inventory (WAI-SR) [109] will be applied to measure the therapeutic alliance between client and e-coach. The 12-item self-report questionnaire covers the three subscales a) agreement on tasks, b) agreement on goals and c) development of an affective bond. For the German Version, internal consistencies between α = .81 and α = .91 were reported for the subscales and internal consistencies between α = .90 and α = .93 for the total score [109, 110]. Participants in IG will complete the WAI-SR at T1 and T3. Additionally, e-coaches will be requested to complete the 10-item therapist version (WAI-SRT, developed by Adam O. Horvath, http://wai.profhorvath.com/) at T1 and T3. This will allow us to compare how the therapeutic relationship is experienced by client and by e-coach, to gain a differentiated and comprehensive picture of the experienced working alliance. The WAI-SR and the WAI-SRT were adapted in wording for the current study investigating therapeutic alliance in guided IMIs. The items were changed to refer to e-coaches instead of therapists and to online-trainings instead of therapy. Additionally, the Technological Alliance Inventory - Online Therapy (TAI-OT) will be administered to assess the technological alliance between client and the online-intervention. The TAI-OT is a new self-report questionnaire developed by Labpsitec (http://www.labpsitec.uji.es/esp/index.php) consisting of 12 items and measures the degree to which the online-program is perceived as helpful in achieving therapeutic goals.

#### Intervention satisfaction

Intervention satisfaction will be assessed using a German Version of the Client Satisfaction Questionnaire (CSQ-8 [111]; German Version: ZUF-8 [112]), specifically adapted for assessing patient satisfaction with IMIs (CSQ-I) [113]. The CSQ-8 is a self-report questionnaire consisting of 8 items characterised by high internal consistency (α = 0.93) [111]. The adapted German version CSQ-I has been validated for the assessment of patient satisfaction with IMIs and is characterised by equally high internal consistency [113]. CSQ-I will be applied to assess satisfaction with online-trainings in IG. An adapted version of the CSQ-I will be applied in CG to evaluate satisfaction with information material.

#### Side effects of psychotherapy

Side effects of psychotherapy will be assessed with the Inventory for the Assessment of Negative Effects of Psychotherapy (INEP). The INEP records whether any negative changes, which are experienced during or after the treatment in the social and/or work environment, are attributed on the psychotherapeutic intervention [114]. In this trial, an adapted 22-item version covering possible negative effects associated specifically with online-trainings (e.g. concerns about data protection) is applied.

Additionally, an open question will be included for qualitative assessments of negative side effects of IMIs. Participants will describe experienced negative events and side effects, their time of beginning, their frequency, and their duration. Two further questions rate the negative impact of these events in the past and at present time.

### Other assessments

#### Medical condition

Participants will be requested to visit a general practitioner and return a standardized formula for clarification of medical condition. This procedure is applied to ensure participants suffering from severe medical conditions receive appropriate medical care. Since the aim of the present study is to evaluate effectiveness (not efficacy) of online-trainings in standard care, the GP visit as well as the return of the formula is left to the responsibility of the participant. Further, the presence of diabetes mellitus will be assessed using a self-report item as part of the psycho-diagnostic assessment for the IMI program.

#### Covariates

As potential moderating variables, demographic information (e.g. gender, age, education), information about the agricultural farm (e.g. farm size, area cultivated, number of workers), and about the situation of the entrepreneurial family (e.g. financial situation, number of relatives living and working together, general work load) will be recorded at baseline. Further, a variety of predictors (e.g. personality, prior experience of violence and aggression, childhood experiences) will be included to assess relevant factors for development of depressive symptomology.

### Procedure on suicidal ideations

Suicidal ideation will be screened using PHQ-9 in screening assessment and QIDS-SR16 for further assessment points (T0-T5). A score ≥ 1 on suicide item of PHQ-9 (“Thoughts that you would be better off dead or of hurting yourself in some way?”) or QIDS-SR16 (QIDS-SR16 Item = 1: “I feel that life is empty or wonder if it’s worth living”, QIDS-SR16 Item = 2: “I think of suicide or death several times a week for several minutes”, QIDS-SR16 Item = 3: “I think of suicide or death several times a day in some detail, or I have made specific plans for suicide or have actually tried to take my life”), leads to BDI-II suicide item [72]. A score ≥ 1 on BDI-II suicide item results in a standardized suicide protocol adapted from prior trials [115, 116].

Participants receive an online information letter with detailed information on available health services and the advice to seek professional help if symptoms increase. The wording of the online information letter is adapted in emphasis, depending on the severity of the indicated suicidality (BDI-II Item = 1: “I have thoughts of killing myself, but I would not carry them out”, BDI-II Item = 2: “I would like to kill myself”, BDI-II-Item = 3: “I would kill myself if I had the chance”). This procedure will be administered on all assessment points and independent of study inclusion.

Eligible participants will be asked to return a signed non-suicide contract as part of screening and baseline (T0), if they are able to distance themselves from suicidal ideation. Participants are informed that the IMI program is not provided as intervention for problems accompanied by persisting suicidal ideation.

For BDI > 1, a detailed clarification of self-endangerment will be performed for all screened participants independent of study inclusion status and assessment point. A telephone contact will be announced via e-mail in case of not returning a given text module or sending an informal answer in response to the online information letter within 48 h of undergoing the assessment point. If self-endangerment remains unclear, psychotherapists (at least) in training will contact the participant via telephone and initiate further actions. Further actions will be determined depending on perceived endangerment. If participant remains not contactable for further 48 h, the police department will be informed. All steps are closely monitored and supervised by a licenced psychological psychotherapist.

### Statistical analyses

#### Clinical analyses

All statistical analyses will be performed based on intention-to-treat (ITT) principle. Patterns of missing data will be examined and analyses will be corrected for missing data by applying multivariate imputation according to Van Buuren and Groothuis-Oudshoorn [117]. For those who substantially completed the intervention (at least 80% of the modules) additional per-protocol (PP) analyses will be conducted. Group difference between pre- and post-assessment nine weeks after randomization (T1) in primary outcome will be assessed using generalized linear modelling. Between-group effect size will be reported using standardized mean differences and 95% CIs at all measurement points. Reliable change index (RCI) and number needed to treat (NNT) will be calculated to determine clinical significance. Analysis of long-term effects will be adjusted to the data structure by using e.g. robust estimation of standard errors or mixed effect modelling. Group differences in depression onset will be assessed by calculation of a Poisson regression model. Incidence of clinical depression will be compared in IG and CG by calculation of incidence rate ratio (IRR). Other secondary outcomes like perceived stress, sleep quality, and quality of life will be analysed similarly as primary outcome. Additionally, moderation and mediation analyses will be performed.

#### Economic evaluation

The health-economic evaluation will involve a combination of a cost-effectiveness analysis (CEA) and a cost-utility analysis (CUA). The economic evaluation will be performed from a societal perspective (all relevant costs) and a public health care perspective (only direct medical costs) with a time horizon of 36 months. In the cost-effectiveness analysis, the incremental cost-effectiveness ratio (ICER) will be based on the incremental costs per unit of effect gained (e.g. reliably improved case based on RCI). The corresponding equation is ICER = (Costs_IG_– Costs_CG_)/(Effects_IG_– Effects_CG_), where Costs are the annual per-participant costs and Effects are the unit of effect in IG and CG. In the CUA, the ICER will be expressed as incremental costs per quality adjusted life year (QALY) gained as based on the AQoL-8D. Sampling uncertainty in the ICER will be handled using nonparametric bootstrapping by resampling on patient-level. In addition, confidence intervals for ICERs will be obtained by bootstrapped quantiles. The bootstrapped ICERs will be plotted in a cost-effectiveness plane where the horizontal axis reflects differences in effects and the vertical axis differences in costs. The bootstrapped ICERs will also be shown in a cost-effective acceptability curve disclosing the probability that the intervention is cost-effective for a range of willingness-to-pay ceilings. To test the robustness of the base-case findings, multi-way sensitivity analyses will be done. Several assumptions made in the base-case scenario (e.g. about cost prices and volumes) will be changed to assess their impact on the ICERs.

## Discussion

This study will be the first to investigate the clinical and cost-effectiveness of a tailored, guided IMI program for the prevention of depression and the reduction in depressive symptom severity in green professions.

The proposed study is characterized by several strengths. First, participants are provided with an IMI program with the option of engaging in an IMI tailored to individual needs. This might enlarge the reach of preventive IMIs by avoiding the exclusive label of depression and associated stigma. Further, this participatory and patient-centred approach might improve therapy motivation, if symptomatology has a clinical burden and psychotherapy is indicated. Also, the addressing of beliefs and needs of participants in form of a tailored and complaint-centred IMI program might strengthen treatment credibility. Intrinsic motivation and treatment credibility have been shown to positively relate to treatment adherence [34, 35]. Thereby, this complaint-based approach might be able to improve the overall impact of preventive internet-based interventions in routine health care by a) increasing the uptake of a preventive IMI program and b) by improving adherence to the IMI program. A large-scale dissemination is especially in prevention context highly desirable to reduce overall depression burden in population as well as associated health care costs. If proven effective, further studies could evaluate the proposed IMI program regarding its reach compared to single IMIs build, labelled and advertised solely as a depression prevention intervention. Second, we will be able to investigate long-term effectiveness of the IMI program. Previous research yielded few and inconsistent results regarding long-term efficacy of psychological interventions for subthreshold depression [18] and IMIs in particular [26]. This study will not only evaluate positive treatment effects, but also possible side effects of the IMI program. Since side effects of preventive IMIs in both short and long terms have not been sufficiently explored yet, this study will be able to provide a detailed understanding about positive and negative treatment effects of the proposed IMI program. Third, we will be able to conduct long-term cost-effectiveness analyses over a 36-month period. Evaluation of a guided IMI for depression prevention has already been shown to yield acceptable cost-effectiveness in preventing onset of clinical depression in people with subthreshold depression over a 12-month evaluation period [56]. However, cost-effectiveness might be less pronounced over a longer follow-up period [56] since results of a first meta-analytic review indicate that effectiveness of psychological interventions for prevention of depression might decline with longer follow-up periods [19]. The present study aims to yield first results regarding cost-effectiveness of an IMI program for prevention of depression over an extensive follow-up period of 36 months to close this knowledge gap. Forth, as a unique feature of the present trial, (cost)effectiveness of an IMI program provided in routine health care provided by the service provider GET.ON will be examined. Based on the pragmatic setting, this effectiveness trial is characterized by high external validity and generalizability regarding the target group of green professions [118, 119]. The efficacy of the employed IMIs in reduction of depressive symptomology has already been demonstrated in previous studies [23–25, 48–55]. For the present trial, the IMI program has been adapted to the context of green professions by the service provider to evaluate effectiveness in routine health care of the target group. Effectiveness and cost-effectiveness is especially interesting against the background of ongoing implementation of this IMI program into standard health care. Thereby, the present trial evaluates clinical and cost-effectiveness of a prospective supply situation in German preventive health care.

Limitations of the present study encompass that, firstly, intervention and study dropout must be considered against the background of such an extensive follow-up period to generate reliable results regarding long-term (cost)effectiveness of the proposed IMI program. Especially participants of the CG are expected to be at risk for study dropout, since they do not get access to the IMI program during the follow-up period of 36 months. To improve study adherence, incentives for completion of online-assessments and standardized reminding procedures are applied. To reduce intervention dropout, IMIs have been adapted in content for green professions and therapeutic guidance is deployed. Dropout can be reduced by providing therapeutic guidance [69]. Additionally, the high number of measurement points could lead to repetition effects like automatic response tendencies, which might limit the informative value of the measurement. Second, representativeness of the investigated sample and generalizability to the target population of green professions might be limited to some degree because of two aspects; for one, high comorbidity between chronic pain and depressive disorders [120] might lead to a substantial proportion of participants choosing participation in the related trial addressing chronic pain (PACT-A, (Terhorst Y, Braun L, Titzler I, Buntrock C, Freund J, Thielecke J, et al.: Effectiveness and cost-effectiveness of an acceptance and commitment therapy to improve chronic pain-related disability in green professions (PACT-A): study protocol of a 36-month follow up pragmatic randomized controlled trial, in preparation)) instead of the present trial, possibly resulting in an underrepresentation of participants with chronic pain in the present study sample. On the other hand, internet access as well as access to an appropriate device (e.g. computer, tablet, smartphone) and basic knowledge of its functioning are an elementary prerequisite for participation in the present trial and the usage of the IMI program. Both might be limited, especially in rural areas, which might systematically lead to the exclusion of persons who have either insufficient internet connection nor the means or the knowledge to work with the required electronic devices. Thus, the present trial will only be representative for the population with the possibility to access the intervention at hand. This limitation is not specific to IMIs but a general limitation of RCTs that are always only representative to the populations who can access the intervention (e.g. on site-interventions only for those who are sufficiently mobile). Third, presence of clinical depression will not be assessed with standardized diagnostic procedures at baseline or follow-up. Since QIDS-SR16 has been shown to have high predictive validity as a screening instrument for MDD [77], this instrument was applied to identify possible cases of MDD. Fourth, no maximum level of depressive symptom severity was defined as eligibility criteria. Therefore, possible cases of manifest clinical depression might be included in the present trial. The present study reflects service reality in this that people with manifest mental disorders can make use of preventive interventions in the absence of structured diagnostic procedures. We will perform categorical analyses of acquired data to estimate percentage of possible cases of illness included. This might provide first insights into actual utilization and impact of preventive interventions in routine health care regarding (lacking) differentiation between subthreshold and manifest clinical depression.

Internet-based preventive interventions have the potential to improve the general care situation substantially, specifically in rural areas, provided that the proposed health care offer encounters reasonable acceptance and utilization in green professions. The proposed tailored prevention approach is thought to improve dissemination of preventive interventions aiming to overcome depressive symptoms or risk factors for development of depression. Such prevention approaches can contribute to reduction of chronification and overall depression burden in green professions. This in turn can result in decrease of sick days, incapacity to work and early retirement, and thus a substantial cost reduction in the health care sector of green professions. If proven effective, implementation of the proposed IMI program into routine health care of green professions will have a large public health impact.

## Trial status

The study is currently ongoing. Recruitment started in January 2018 and was concluded in April 2019.

## Data Availability

Not applicable.

## Abbreviations

AQoL-8D: Assessment of Quality of Life
AUDIT: Alcohol Use Disorder Identification Test
BDI-II: Beck Depression Inventory
BPD: Bipolar disorder
CEA: Cost-effectiveness analysis
CG: Control group
CIDI: Composite International Diagnosis Interview
CONSORT: Consolidated Standards of Reporting Trials
CPG: Chronic Pain Grade Questionnaire
CSQ-8: Client Satisfaction Questionnaire
CSQ-I: Client Satisfaction Questionnaire adapted to internet-based interventions
CUA: Cost-utility analysis
GAD-7: Generalized Anxiety Disorder Screener
ICC: Intraclass Correlation Coefficient
ICER: Incremental cost-effectiveness ratio
IG: Intervention group
IMI: Internet- and mobile-based intervention
IMMPACT: Initiative on Methods, Measurement and Pain Assessment in Clinical Trials
INEP: Inventory for the Assessment of Negative Effects of Psychotherapy
IRR: Incidence Rate Ratio
ISI: Insomnia Severity Index
ITT: Intention-to-treat
MBI-GS: Maslach Burnout Inventory - General Survey
MDD: Major depressive disorder
MDE: Major depressive episode
MPI: Multidimensional Pain Interference Scale
NNT: Number needed to treat
PAS: Panic and Agoraphobia Scale
PHQ-9: Patient Health Questionnaire
PP: Per-protocol
PSS-10: Perceived Stress Scale
QALY: Quality adjusted life year
QIDS-SR16: Quick Inventory Depressive Symptomology
RCI: Reliable Change Index
RCT: Randomized controlled trial
RR: Risk Ratio
SCID: Structured Clinical Interview for DSM-IV-TR Axis I Disorders
SPE: Subjective Prognostic Employment Scale
SVLFG: Social Insurance for Agriculture, Forestry and Horticulture
TAI-OT: Technological Alliance Inventory - Online Therapy
TAU: Treatment as usual
TiC-P: Trimbos Institute and Institute of Medical Technology Questionnaire for Costs Associated with Psychiatric Illness
WAI-SR: Working Alliance Inventory - Short Revised
WAI-SRT: Working Alliance Inventory - Short Revised - Therapist

## References

[1] Kessler RC, Chiu WT, Demler O, Walters EE (2005). Prevalence, severity, and comorbidity of 12-month DSM-IV disorders in the National Comorbidity Survey Replication. Arch Gen Psychiatry.

[2] Kessler RC, Berglund P, Demler O, Jin R, Merikangas KR, Walters EE (2005). Lifetime prevalence and age-of-onset distributions’ of DSM-IV disorders in the National Comorbidity Survey Replication. Arch Gen Psychiatry.

[3] Bromet E, Andrade LH, Hwang I, Sampson NA, Alonso J, de Girolamo G (2011). Cross-national epidemiology of DSM-IV major depressive episode. BMC Med.

[4] Kessler RC, Merikangas KR, Wang PS (2007). Prevalence, comorbidity, and service utilization for mood disorders in the United States at the beginning of the twenty-first century. Annu Rev Clin Psychol.

[5] Ferrari AJ, Charlson FJ, Norman RE, Patten SB, Freedman G, Murray CJL (2013). Burden of depressive disorders by country, sex, age, and year: findings from the global burden of disease study 2010. PLoS Med.

[6] Greenberg PE, Fournier A-A, Sisitsky T, Pike CT, Kessler RC (2015). The economic burden of adults with major depressive disorder in the United States (2005 and 2010). J Clin Psychiatry.

[7] Simkin S, Hawton K, Fagg J, Malmberg A (1998). Stress in farmers: a survey of farmers in England and Wales. Occup Environ Med.

[8] Onwuameze OE, Paradiso S, Peek-Asa C, Donham KJ, Rautiainen RH (2013). Modifiable risk factors for depressed mood among farmers. Ann Clin Psychiatry.

[9] Krawczyk N, Meyer A, Fonseca M, Lima J (2014). Suicide mortality among agricultural Workers in a Region with Intensive Tobacco Farming and use of pesticides in Brazil. J Occup Environ Med.

[10] Stallones L, Beseler C (2002). Pesticide Poisioning and depressive symptoms among farm residents. Ann Epidemiol.

[11] Charlton J, Kelly S, Dunnell K, Evans B, Jenkins R (1993). Suicide deaths in England and Wales: trends in factors associated with suicide deaths. Popul Trends.

[12] Hawton K, Fagg J, Simkin S, Harriss L, Malmberg A, Smith D (1999). The geographical distribution of suicides in farmers in England and Wales. Soc Psychiatry Psychiatr Epidemiol.

[13] Stallones L (1990). Suicide mortality among Kentucky farmers, 1979-1985. Suicide Life-Threatening Behav.

[14] McIntosh WL, Spies E, Stone DM, Lokey CN, Trudeau A-RT, Bartholow B (2016). Suicide Rates by Occupational Group — 17 States, 2012. MMWR Morb Mortal Wkly Rep.

[15] Gunderson P, Donner D, Nashold R, Salkowicz L (1970). The epidemiology of suicide among farm residents or workers in five north-central states, 1980-1988. Am J Prev Med.

[16] Miller K, Burns C (2008). Suicides on farms in South Australia, 1997-2001. Aust J Rural Health.

[17] Page AN, Fragar LJ (2002). Suicide in Australian farming, 1988-1997. Aust N Z J Psychiatry..

[18] Cuijpers P, Koole SL, Van Dijke A, Roca M, Li J, Reynolds CF (2014). Psychotherapy for subclinical depression: meta-analysis. Br J Psychiatry.

[19] van Zoonen K, Buntrock C, Ebert DD, Smit F, Reynolds CF, Beekman ATF (2014). Preventing the onset of major depressive disorder: a meta-analytic review of psychological interventions. Int J Epidemiol.

[20] Bundespsychotherapeutenkammer (2018). Ein Jahr nach der Reform der Psychotherapie-Richtlinie. Wartezeiten 2018.

[21] Spek V, Nyklíček I, Smits N, Cuijpers P, Riper H, Keyzer J (2007). Internet-based cognitive behavioural therapy for subthreshold depression in people over 50 years old: a randomized controlled clinical trial. Psychol Med.

[22] Imamura K, Kawakami N, Furukawa TA, Matsuyama Y, Shimazu A, Umanodan R (2014). Effects of an internet-based cognitive behavioral therapy (iCBT) program in manga format on improving subthreshold depressive symptoms among healthy workers: a randomized controlled trial. PLoS One.

[23] Buntrock C, Ebert D, Lehr D, Riper H, Smit F, Cuijpers P (2015). Effectiveness of a web-based cognitive Behavioural intervention for subthreshold depression: pragmatic randomised controlled trial. Psychother Psychosom.

[24] Buntrock C, Ebert DD, Lehr D, Smit F, Riper H, Berking M (2016). Effect of a web-based guided self-help intervention for prevention of major depression in adults with subthreshold depression: a randomized clinical trial. Jama..

[25] Ebert DD, Buntrock C, Lehr D, Smit F, Riper H, Baumeister H (2018). Effectiveness of web- and Mobile-based treatment of subthreshold depression with adherence-focused guidance: a single-blind randomized controlled trial. Behav Ther.

[26] Zhou T, Li X, Pei Y, Gao J, Kong J (2016). Internet-based cognitive behavioural therapy for subthreshold depression: a systematic review and meta-analysis. BMC Psychiatry..

[27] Ebert DD, Cuijpers P, Muñoz RF, Baumeister H (2017). Prevention of mental health disorders using internet- and Mobile-based interventions: a narrative review and recommendations for future research. Front Psychiatry.

[28] Sander L, Rausch L, Baumeister H (2016). Effectiveness of internet-based interventions for the prevention of mental disorders: a systematic review and meta-analysis. JMIR Ment Heal.

[29] Ebert DD, Van Daele T, Nordgreen T, Karekla M, Compare TA, Zarbo C (2018). Internet and mobile-based psychological interventions: applications, efficacy and potential for improving mental health. Eur Psychol.

[30] Titov N, Dear BF, Staples LG, Terides MD, Karin E, Sheehan J (2015). Disorder-specific versus transdiagnostic and clinician-guided versus self-guided treatment for major depressive disorder and comorbid anxiety disorders: a randomized controlled trial. J Anxiety Disord.

[31] Titov N, Andrews G, Johnston L, Robinson E, Spence J (2010). Transdiagnostic internet treatment for anxiety disorders: a randomized controlled trial. Behav Res Ther.

[32] Karyotaki E, Kleiboer A, Smit F, Turner DT, Pastor AM, Andersson G (2015). Predictors of treatment dropout in self-guided web-based interventions for depression: an ‘ individual patient data ’ meta-analysis. Psychol Med.

[33] Melville KM, Casey LM, Kavanagh DJ (2010). Dropout from internet-based treatment for psychological disorders. Br J Clin Psychol.

[34] Alfonsson S, Johansson K, Uddling J, Hursti T (2017). Differences in motivation and adherence to a prescribed assignment after face-to-face and online psychoeducation: an experimental study. BMC Psychol.

[35] Alfonsson S, Olsson E, Hursti T (2016). Motivation and treatment credibility predicts dropout, treatment adherence, and clinical outcomes in an internet-based cognitive behavioral relaxation program: a randomized controlled trial. J Med Internet Res.

[36] Ebert DD, Berking M, Cuijpers P, Lehr D, Pörtner M, Baumeister H (2015). Increasing the acceptance of internet-based mental health interventions in primary care patients with depressive symptoms. A randomized controlled trial. J Affect Disord.

[37] Baumeister H, Seifferth H, Lin J, Nowoczin L, Lüking M, Ebert DD (2015). Impact of an acceptance facilitating intervention on patients’ acceptance of internet-based pain interventions: a randomized controlled trial. Clin J Pain.

[38] Baumeister H, Nowoczin L, Lin J, Seifferth H, Seufert J, Laubner K (2014). Impact of an acceptance facilitating intervention on diabetes patients’ acceptance of internet-based interventions for depression: a randomized controlled trial. Diabetes Res Clin Pract.

[39] Lin J, Faust B, Ebert DD, Krämer L, Baumeister H (2018). A web-based acceptance-facilitating intervention for identifying patients’ acceptance, uptake, and adherence of internet- and mobile-based pain interventions: randomized controlled trial. J Med Internet Res.

[40] Wrigley S, Jackson H, Judd F, Komiti A (2005). Role of stigma and attitudes toward help-seeking from a general practitioner for mental health problems in a rural town. Aust N Z J Psychiatry.

[41] Jones AR, Cook TM, Wang J (2011). Rural-urban differences in stigma against depression and agreement with health professionals about treatment. J Affect Disord.

[42] Moher D, Hopewell S, Schulz KF, Montori V, Gotzsche PC, Devereaux PJ (2010). CONSORT 2010 explanation and elaboration: updated guidelines for reporting parallel group randomised trials. BMJ..

[43] Schulz KF, Altman DG, Moher D (2010). CONSORT 2010 statement: updated guidelines for reporting parallel group randomised trials. BMJ..

[44] Zwarenstein M, Treweek S, Gagnier JJ, Altman DG, Tunis S, Haynes B (2008). Improving the reporting of pragmatic trials: an extension of the CONSORT statement. BMJ..

[45] Proudfoot J, Klein B, Barak A, Carlbring P, Cuijpers P, Lange A (2011). Establishing guidelines for executing and reporting internet intervention research. Cogn Behav Ther.

[46] Chan A-W, Tetzlaff JM, Altman DG, Laupacis A, Gøtzsche PC, Krleža-Jerić K (2013). SPIRIT 2013 statement: defining standard protocol items for clinical trials. Ann Intern Med.

[47] Chan A-W, Tetzlaff JM, Gøtzsche PC, Altman DG, Mann H, Berlin JA (2013). SPIRIT 2013 explanation and elaboration: guidance for protocols of clinical trials. BMJ..

[48] Nobis S, Lehr D, Ebert DD, Baumeister H, Snoek F, Riper H (2015). Efficacy of a web-based intervention with Mobile phone support in treating depressive symptoms in adults with type 1 and type 2 diabetes: a randomized controlled trial. Diabetes Care.

[49] Ebert DD, Nobis S, Lehr D, Baumeister H, Riper H, Auerbach RP (2017). The 6-month effectiveness of internet-based guided self-help for depression in adults with type 1 and 2 diabetes mellitus. Diabet Med.

[50] Ebert DD, Heber E, Berking M, Riper H, Cuijpers P, Funk B (2016). Self-guided internet-based and mobile-based stress management for employees: results of a randomised controlled trial. Occup Environ Med.

[51] Ebert DD, Berking M, Thiart H, Riper H, Laferton JAC, Cuijpers P (2015). Restoring depleted resources: efficacy and mechanisms of change of an internet-based unguided recovery training for better sleep and psychological detachment from work. Health Psychol.

[52] Boß L, Lehr D, Schaub MP, Castro RP, Riper H, Berking M (2018). Efficacy of a web-based intervention with and without guidance for employees with risky drinking: results of a three-arm randomized controlled trial. Addiction..

[53] Heber E, Lehr D, Ebert DD, Berking M, Riper H (2016). Web-based and Mobile stress management intervention for employees: a randomized controlled trial. J Med Internet Res.

[54] Thiart H, Lehr D, Ebert DD, Berking M, Riper H (2015). Log in and breathe out: internet-based recovery training for sleepless employees with work-related strain – results of a randomized controlled trial. Scand J Work Environ Health.

[55] Ebert DD, Lehr D, Heber E, Riper H, Cuijpers P, Berking M (2016). Internet- and mobile-based stress management for employees with adherence-focused guidance: efficacy and mechanism of change. Scand J Work Environ Health.

[56] Buntrock C, Berking M, Smit F, Lehr D, Nobis S, Riper H (2017). Preventing depression in adults with subthreshold depression: health-economic evaluation alongside a pragmatic randomized controlled trial of a web-based intervention. J Med Internet Res.

[57] Nobis S, Ebert DD, Lehr D, Smit F, Buntrock C, Berking M (2018). Web-based intervention for depressive symptoms in adults with types 1 and 2 diabetes mellitus: a health economic evaluation. Br J Psychiatry.

[58] Ebert DD, Kählke F, Buntrock C, Berking M, Smit F, Heber E (2018). A health economic outcome evaluation of an internet-based mobile-supported stress management intervention for employees. Scand J Work Environ Health.

[59] Thiart H, Ebert DD, Lehr D, Nobis S, Buntrock C, Berking M (2016). Internet-based cognitive behavioral therapy for insomnia: a health economic evaluation. Sleep..

[60] Mohr DC, Cuijpers P, Lehman K (2011). Supportive accountability: a model for providing human support to enhance adherence to eHealth interventions. J Med Internet Res.

[61] Lazarus RS, Folkman S (1987). Transactional theory and research on emotions and coping. Eur J Pers.

[62] Buntrock C, Ebert DD, Lehr D, Cuijpers P, Riper H, Smit F (2014). Evaluating the efficacy and cost-effectiveness of web-based indicated prevention of major depression: design of a randomised controlled trial. BMC Psychiatry..

[63] Nobis S, Lehr D, Ebert DD, Berking M, Heber E, Baumeister H (2013). Efficacy and cost-effectiveness of a web-based intervention with mobile phone support to treat depressive symptoms in adults with diabetes mellitus type 1 and type 2: design of a randomised controlled trial. BMC Psychiatry..

[64] Thiart H, Lehr D, Ebert DD, Sieland B, Berking M, Riper H (2013). Log in and breathe out: efficacy and cost-effectiveness of an online sleep training for teachers affected by work-related strain - study protocol for a randomized controlled trial. Trials..

[65] Ebert DD, Lehr D, Smit F, Zarski AC, Riper H, Heber E (2014). Efficacy and cost-effectiveness of minimal guided and unguided internet-based mobile supported stress-management in employees with occupational stress: a three-armed randomised controlled trial. BMC Public Health.

[66] Ebenfeld L, Kleine Stegemann S, Lehr D, Ebert DD, Jazaieri H, van Ballegooijen W (2014). Efficacy of a hybrid online training for panic symptoms and agoraphobia: study protocol for a randomized controlled trial. Trials..

[67] Schaub MP, Blankers M, Lehr D, Boss L, Riper H, Dekker J (2016). Efficacy of an internet-based self-help intervention to reduce co-occurring alcohol misuse and depression symptoms in adults: study protocol of a three-arm randomised controlled trial. BMJ Open.

[68] Bouwmans C, De Jong K, Timman R, Zijlstra-Vlasveld M, Van der Feltz-Cornelis C, Tan SS (2013). Feasibility, reliability and validity of a questionnaire on healthcare consumption and productivity loss in patients with a psychiatric disorder (TiC-P). BMC Health Serv Res.

[69] Richards D, Richardson T (2012). Computer-based psychological treatments for depression: a systematic review and meta-analysis. Clin Psychol Rev.

[70] Löwe B, Kroenke K, Herzog W, Gräfe K (2004). Measuring depression outcome with a brief self-report instrument: sensitivity to change of the patient health questionnaire (PHQ-9). J Affect Disord.

[71] Erbe D, Eichert HC, Rietz C, Ebert DD (2016). Interformat reliability of the patient health questionnaire: validation of the computerized version of the PHQ-9. Internet Interv.

[72] Kühner C, Bürger C, Keller F, Hautzinger M (2007). Reliabilität und Validität des revidierten Beck- Depressionsinventars (BDI-II). Befunde aus deutschsprachigen Stichproben. Nervenarzt..

[73] Von Korff M, Ormel J, Keefe FJ, Dworkin SF (1992). Grading the severity of chronic pain. Pain..

[74] Klasen BW, Hallner D, Schaub C, Willburger R, Hasenbring M (2004). Validation and reliability of the German version of the Chronic Pain Grade questionnaire in primary care back pain patients. Psycho-Social-Medicine.

[75] Rush AJ, Trivedi MH, Ibrahim HM, Carmody TJ, Arnow B, Klein DN (2003). The 16-item quick inventory of depressive symptomatology (QIDS), clinician rating (QIDS-C), and self-report (QIDS-SR): a psychometric evaluation in patients with chronic major depression. Biol Psychiatry.

[76] Jacobson NS, Truax P (1991). Clinical significance: a statistical approach to defining meaningful change in psychotherapy research. J Consult Clin Psychol.

[77] Lamoureux BE, Linardatos E, Fresco DM, Bartko D, Logue E, Milo L (2010). Using the QIDS-SR16 to identify major depressive disorder in primary care medical patients. Behav Ther.

[78] Kessler RC, Üstün TB (2004). The world mental health (WMH) survey initiative version of the World Health Organization (WHO) composite international diagnostic interview (CIDI). Int J Methods Psychiatr Res.

[79] Kessler RC, Farley PA, Gruber M, Harshaw Q, Jewell MA, Sampson N (2010). PMH72 concordance of computerized self-report measures of DSM-IV-TR, Mood and Anxiety Disorders Compared To Gold Standard Clinical Assessments in Primay Care. Value Heal.

[80] Auerbach RP, Mortier P, Bruffaerts R, Alonso J, Benjet C, Cuijpers P (2018). WHO world mental health surveys international college student project: prevalence and distribution of mental disorders. J Abnorm Psychol.

[81] Cohen S, Kamarck T, Mermelstein R (1983). A global measure of perceived stress. J Health Soc Behav.

[82] Cohen S, Willliamson GM, Spacapan S, Oskamp S (1988). Perceived stress in a probability sample of the United States. The social psychology of health.

[83] Cohen S, Janicki-Deverts D (2012). Who’s stressed? Distributions of psychological stress in the United States in probability samples from 1983, 2006, and 2009. J Appl Soc Psychol.

[84] Klein EM, Brähler E, Dreier M, Reinecke L, Müller KW, Schmutzer G (2016). The German version of the perceived stress scale – psychometric characteristics in a representative German community sample. BMC Psychiatry.

[85] Bastien CH, Vallières A, Morin CM (2001). Validation of the insomnia severity index as an outcome measure for insomnia research. Sleep Med.

[86] Morin CM, Belleville G, Bélanger L, Ivers H (2011). The insomnia severity index: psychometric indicators to detect insomnia cases and evaluate treatment response. Sleep..

[87] Gagnon C, Belanger L, Ivers H, Morin CM (2013). Validation of the insomnia severity index in primary care. J Am Board Fam Med.

[88] Gerber M, Lang C, Lemola S, Colledge F, Kalak N, Holsboer-Trachsler E (2016). Validation of the German version of the insomnia severity index in adolescents, young adults and adult workers: results from three cross-sectional studies. BMC Psychiatry..

[89] Spitzer RL, Kroenke K, Williams JBW, Löwe B (2006). A brief measure for assessing generalized anxiety disorder: the GAD-7. Arch Intern Med.

[90] Löwe B, Decker O, Müller S, Brähler E, Schellberg D, Herzog W (2008). Validation and standardization of the generalized anxiety disorder screener (GAD-7) in the general population. Med Care.

[91] Bandelow B (1995). Assessing the efficacy of treatments for panic disorder and agoraphobia. II. The panic and agoraphobia scale. Int Clin Psychopharmacol.

[92] Bandelow B, Hajak G, Holzrichter S, Kunert HJ, Rüther E (1995). Assessing the efficacy of treatments for panic disorder and agoraphobia. I. Methodological problems. Int Clin Psychopharmacol.

[93] Dworkin RH, Turk DC, Farrar JT, Haythornthwaite JA, Jensen MP, Katz NP (2005). Core outcome measures for chronic pain clinical trials: IMMPACT recommendations. Pain..

[94] Dworkin RH, Turk DC, Wyrwich KW, Beaton D, Cleeland CS, Farrar JT (2008). Interpreting the clinical importance of treatment outcomes in chronic pain clinical trials: IMMPACT recommendations. J Pain.

[95] Kerns RD, Turk DC, Rudy TE (1985). The west haven-Yale multidimensional pain inventory (WHYMPI). Pain..

[96] Flor H, Rudy TE, Birbaumer N, Streit B, Schugens MM (1990). Zur Anwendbarkeit des west haven-Yale multidimensional pain inventory im deutschen Sprachraum - Daten zur Reliabilität und Validität des MPI-D. Schmerz.

[97] Babor T, Higgins-Biddle JC, Saunders JB, Monteiro MG (2001). AUDIT: the alcohol use disorders identification test: guidelines for use in primary care.

[98] Saunders JB, Aasland OG, Amundsen A, Grant M (1993). Alcohol consumption and related problems among primary health care patients: WHO collaborative project on early detection of persons with harmful alcohol consumption - I. Addiction..

[99] Saunders JB, Aasland OG, Babor TF, Fuente JR, Grant M (1993). Development of the alcohol use disorders identification test (AUDIT): WHO collaborative project on early detection of persons with harmful alcohol consumption - II. Addiction..

[100] Fleming MF, Barry KL, MacDonald R (1991). The alcohol use disorders identification test (AUDIT) in a college sample. Int J Addict.

[101] Hays RD, Merz JF, Nicholas R (1995). Response burden, reliability, and validity of the CAGE, short MAST, and AUDIT alcohol screening measures. Behav Res Methods, Instruments, Comput.

[102] Dybek I, Bischof G, Grothues J, Reinhardt S, Meyer C, Hapke U (2006). The Reliability and Validity of the Alcohol Use Disorders Identification Test (AUDIT) in a German General Practice Population Sample. J Stud Alcohol.

[103] Maslach C, Jackson SE, Leiter MP. Maslach Burnout Inventory. In: Zalaquett CP, Wood RJ, editors. Evaluating Stress: A Book of Resources. 3rd ed. Lanham: The Scarecrow Press; 1997. p. 191–218.

[104] Cillien P, Fischbach A, Mörsdorf A, Scherp E, Schaufeli WB (2006). Maslach Burnout Inventory-General Survey Deutsche Version 1.0 (MBI-GS-D V1.0.).

[105] Richardson J, Iezzi A, Khan MA, Maxwell A (2014). Validity and reliability of the assessment of quality of life (AQoL)-8D multi-attribute utility instrument. Patient..

[106] Mittag O, Raspe H (2003). Eine kurze Skala zur Messung der subjektiven Prognose der Erwerbstätigkeit: Ergebnisse einer Untersuchung an 4279 Mitgliedern der gesetzlichen Arbeiterrentenversicherung zu Reliabilität (Guttman-Skalierung) und Validität der Skala. Rehabilitation..

[107] Drummond MF, Sculpher MJ, Torrance GW, O’Brien BJO, Stoddart GL (2005). Methods for the economic evaluation of health care Programmes.

[108] Bock JO, Brettschneider C, Seidl H, Bowles D, Holle R, Greiner W (2015). Ermittlung standardisierter Bewertungssätze aus gesellschaftlicher Perspektive für die gesundheitsökonomische evaluation. Gesundheitswesen..

[109] Wilmers F, Munder T, Leonhart R, Herzog T, Plassmann R, Barth J (2008). Die deutschsprachige version des working Alliance inventory – short revised (WAI-SR) – Ein schulenübergreifendes, ökonomisches und empirisch validiertes instrument zur Erfassung der therapeutischen Allianz. Klin Diagnostik und Eval.

[110] Munder T, Wilmers F, Leonhart R, Linster HW, Barth J (2010). Working Alliance inventory-short revised (WAI-SR): psychometric properties in outpatients and inpatients. Clin Psychol Psychother.

[111] Attkisson CC, Zwick R (1982). The Client Satisfaction Questionnaire. Eval Program Plann.

[112] Schmidt J, Lamprecht F, Wittmann W (1989). Satisfaction with inpatient management. Development of a questionnaire and initial validity studies. Psychother Psychosom Med Psychol.

[113] Boß L, Lehr D, Reis D, Vis C, Riper H, Berking M (2016). Reliability and validity of assessing user satisfaction with web-based health interventions. J Med Internet Res.

[114] Ladwig I, Rief W, Nestoriuc Y (2014). What are the risks and side effects of psychotherapy? – development of an inventory for the assessment of negative effects of psychotherapy (INEP). Verhaltenstherapie..

[115] Sander L, Paganini S, Lin J, Schlicker S, Ebert DD, Buntrock C (2017). Effectiveness and cost-effectiveness of a guided internet- and mobile-based intervention for the indicated prevention of major depression in patients with chronic back pain – study protocol of the PROD-BP multicenter pragmatic RCT. BMC Psychiatry..

[116] Lin J, Lüking M, Ebert DD, Buhrman M, Andersson G, Baumeister H (2015). Effectiveness and cost-effectiveness of a guided and unguided internet-based acceptance and commitment therapy for chronic pain: study protocol for a three-armed randomised controlled trial. Internet Interv.

[117] Buuren SV, Groothuis-Oudshoorn K. mice: Multivariate Imputation by Chained Equations in R. J Stat Softw. 2011;45. 10.18637/jss.v045.i03.

[118] Singal AG, Higgins PDR, Waljee AK (2014). A primer on effectiveness and efficacy trials. Clin Transl Gastroenterol.

[119] Goldfried MR, Wolfe BE (1998). Toward a more clinically valid approach to therapy research. J Consult Clin Psychol.

[120] Bair MJ, Robinson RL, Katon W, Kroenke K (2003). Depression and pain comorbidity. Arch Intern Med.

